# Movement patterns in tuba playing: comparison of an embouchure dystonia case with healthy professional tuba players using real-time MRI imaging

**DOI:** 10.3389/fneur.2023.1106217

**Published:** 2023-05-10

**Authors:** Robert Nelkenstock, Peter W. Iltis, Dirk Voit, Jens Frahm, Edoardo Passarotto, Eckart Altenmüller

**Affiliations:** ^1^Institut für Musikphysiologie und Musikermedizin, Hochschule für Musik, Theater und Medien, Hannover, Germany; ^2^Department of Kinesiology, Gordon College, Wenham, MA, United States; ^3^Biomedizinische NMR, Max-Planck-Institut für Multidisziplinäre Naturwissenschaften, Göttingen, Germany

**Keywords:** real-time MRI, brass playing, embouchure dystonia, musician’s dystonia, tongue movements, movement disorder, tuba playing

## Abstract

**Introduction:**

Musculoskeletal problems in professional brass musicians are very common and often involve the muscles of the embouchure. In rare cases, embouchure dystonia (EmD), a task-specific movement disorder with a wide symptomatic and phenotypic variability, occurs. Following trumpeters and horn players, professional tuba players with and without EmD have now been studied using the latest real-time MRI technology to better understand the underlying pathophysiology.

**Materials and methods:**

In the present study, the tongue movement patterns of 11 healthy professional artists and one subject suffering from EmD were compared. The tongue position in the anterior, intermediary and posterior oral cavity were converted into pixel positions based on seven previously generated profile lines, using the established software MATLAB. These data allow a structured comparison of tongue movement patterns between the patient and the healthy subjects, as well as between individual exercises. The main focus of the analysis was on an ascending 7-note harmonic series performed in different playing techniques (slurred, tongued, tenuto and staccato).

**Results:**

Playing the ascending harmonics, a noticeable ascending tongue movement could be observed in the anterior part of the oral cavity in healthy tubists. In the posterior region, there was a slight decrease in oral cavity space. In the EmD patient, hardly any movement was observed at the tongue apex, but in the middle and posterior regions of the oral cavity there was an increase in size the higher the tone became. These distinct differences are relevant for the characterization and a better understanding of the clinical presentation of EmD. Concerning different playing techniques, it was apparent, that notes played slurred or staccato resulted in a larger oral cavity when compared to notes played tongued or tenuto, respectively.

**Conclusion:**

By using real-time MRI videos, the tongue movements of tuba players can be clearly observed and analyzed. The differences between healthy and diseased tuba players demonstrate the great effects of movement disorders in a small area of the tongue. In order to better understand the compensation of this motor control dysfunction, further studies should investigate further parameters of tone production in all brass players with a larger number of EmD patients additional to the observed movement patterns.

## Introduction

Making music on a professional level requires a lot of effort from players. Apart from the high psychological pressure (1), especially musculoskeletal problems are common among brass musicians (61%) (2). The individual instrument groups differ in the requirements and the resulting complaints (3, 4).

Developments in real-time MRI (RT-MRI) technology provide new and powerful tools for studying brass performance in musicians. Previously used to study speaking and singing (5), RT-MRI has been extended to describe oral cavity movement patterns in trumpet players (6) and compare tongue movements between different brass instrumentalists (7).

While fundamental studies on tongue movement patterns and comparison between healthy musicians and those suffering from embouchure dystonia (EmD) have been published for horn players (8) and trumpeters (9), low brass instrumentalists (trombone and tuba) have not yet been studied.

A survey of 585 professional brass players shows how susceptible the embouchure of brass musicians is to malfunctions. 60% of the brass musicians reported having had embouchure problems. In addition, 16% of this population stated that they had sick leave due to embouchure problems in their career (10). The average duration of an embouchure crisis was about 41 months (10).

EmD is a task-specific disorder of the muscles of the lower face, tongue, jaw and pharynx, which brass and woodwind players need to produce sounds on their instruments (11). The majority of EmD patients describe that, initially, symptoms were only limited to some select registers or playing techniques (4, 12). As the disease progresses, it is characterized by a drastic decrease in sound and playing quality, severely affecting the professional career (13). Frucht et al. classified all EmD patients according to one of six phenotypes: embouchure tremor, lip-pulling, lip-lock, jaw dystonia, tongue dystonia or meige syndrome (4). Many players resort to compensatory movements to address the muscular symptoms (4). While prior studies have observed the embouchure of dystonia patients from the outside (12), recent studies have shown that the latest high-resolution real-time MRI technology can be used to obtain new results in healthy and diseased musicians (7).

Typically, musician’s dystonia is not accompanied by pain, which distinguishes it from other muscle diseases or injuries (12, 14). The mean age of symptom onset is 36.3 years, occupational absence averages 3 years (4).

The causes of musician’s dystonia are not yet fully understood, but a multifactorial genesis is suspected (14). For instance, it was shown that patients with embouchure dystonia or spasmodic dysphonia had brain network alterations even in absence of dystonia-inducing movements and in brain regions representing other body parts than the affected oral and/or pharyngeal structures (15, 16). These findings are in line with clinical observations, e.g., of coincident Writer’s Cramp and embouchure dystonia (17), hinting at a general vulnerability for developing dystonia.

Even though embouchure dystonia is a movement disorder of neurological origin, psychological stressors have been identified as trigger factors (18), in addition to musculoskeletal overexertion and personality traits such as anxiety and perfectionism (14). From a pathophysiological perspective, a relationship between musician’s dystonia and abnormal somatosensory mapping, or sensorimotor hyperactivity, is suspected (19, 20).

2,626 patients with diagnosed musician’s dystonia are living in Germany, which means a prevalence of 32.2/1mil. in the total German population (21). Whereas only about 0.06% of the total German population suffer from various dystonic disorders (21), a significantly higher prevalence (1%–2%) can be assumed for professional musicians (3, 13).

Previous therapeutic approaches, such as behavioral therapy or combinations of pharmacological and pedagogical methods, have proven to be only moderately effective and are still in the early stages of development (14). While botulinum toxin therapy provides promising results for many types of dystonia, no successful treatment strategy has been demonstrated with respect to EmD (22). Basic information about the disease is still needed to further develop therapeutic and prevention strategies (14).

In a pilot study by Iltis et al. (7), all four brass instruments (trumpet, horn, trombone and tuba) were investigated using real-time MRI (7). It was found that the different instrument groups have their own distinct movement patterns (7). These pilot results have already been reviewed for the horn (8) and the trumpet (9) with larger groups of professional musicians. This study will provide the previously missing results for the tuba, applying an identical methodological approach to quantify the movement trajectories.

The aim of the study is therefore to obtain reference data on common playing techniques of healthy tubists and to compare these data with a case of a patient suffering from EmD.

## Materials and methods

### Subjects

Eleven professional, healthy tuba players (mean age 26.4 years) without embouchure problems and one patient suffering from EmD (23 years), selected by an expert with 30 years of experience in diagnosis and treatment of EmD (author EA), participated in the study. Most of the professional players had already completed their studies at a music university and are now active in professional German orchestras or as freelance musicians. Four of the healthy players are in the advanced stages of their tuba studies at the music universities in Hannover and Karlsruhe, which are ranked among the best in Europe. All of them have already completed far between 18,000 and 36,000 h of cumulative live practice time, allowing for the assumption that the movement patterns have already become consolidated and automated (23). The subjects had no neurological problems or other diseases that could affect the outcome of the study. Detailed information about all subjects can be found in Table 1. All subjects gave written consent for the MRI examination, which was performed at the Max Planck Institute for Multidisciplinary Sciences in Göttingen, as well as for processing of the data and scientific publication. The Ethics Committee of the Hannover Medical School approved the study.

**Table 1:** Detailed information about the subjects.

Subj.	Health status	Age / years	Gender	Professional status	Month since diagnosis	Symptoms	Daily practice hours
1	H	23	M	Student	–	–	7 h
2	H	25	M	Professional	–	–	1 h
3	H	22	F	Student	–	–	5 h
4	H	22	M	Student	–	–	n.a.
5	H	30	M	Professional	–	–	8 h
6	H	24	M	Student	–	–	4 h
7	H	29	M	Professional	–	–	6 h
8	H	27	M	Professional	–	–	4 h
9	H	27	M	Professional	–	–	4 h
10	H	29	M	Professional	–	–	4 h
11	H	32	M	Professional	–	–	6 h
12	D	23	M	Student	24 months	ED severe problems when tonguing. Reduced control of the tongue attack and compensatory tension of embouchure and consequently abnormal fatiguability of embouchure	4 h prior to ED/Now, 0,5 h

Subject 12 was the patient with embouchure dystonia.

#### Instrument

**Figure 1:**
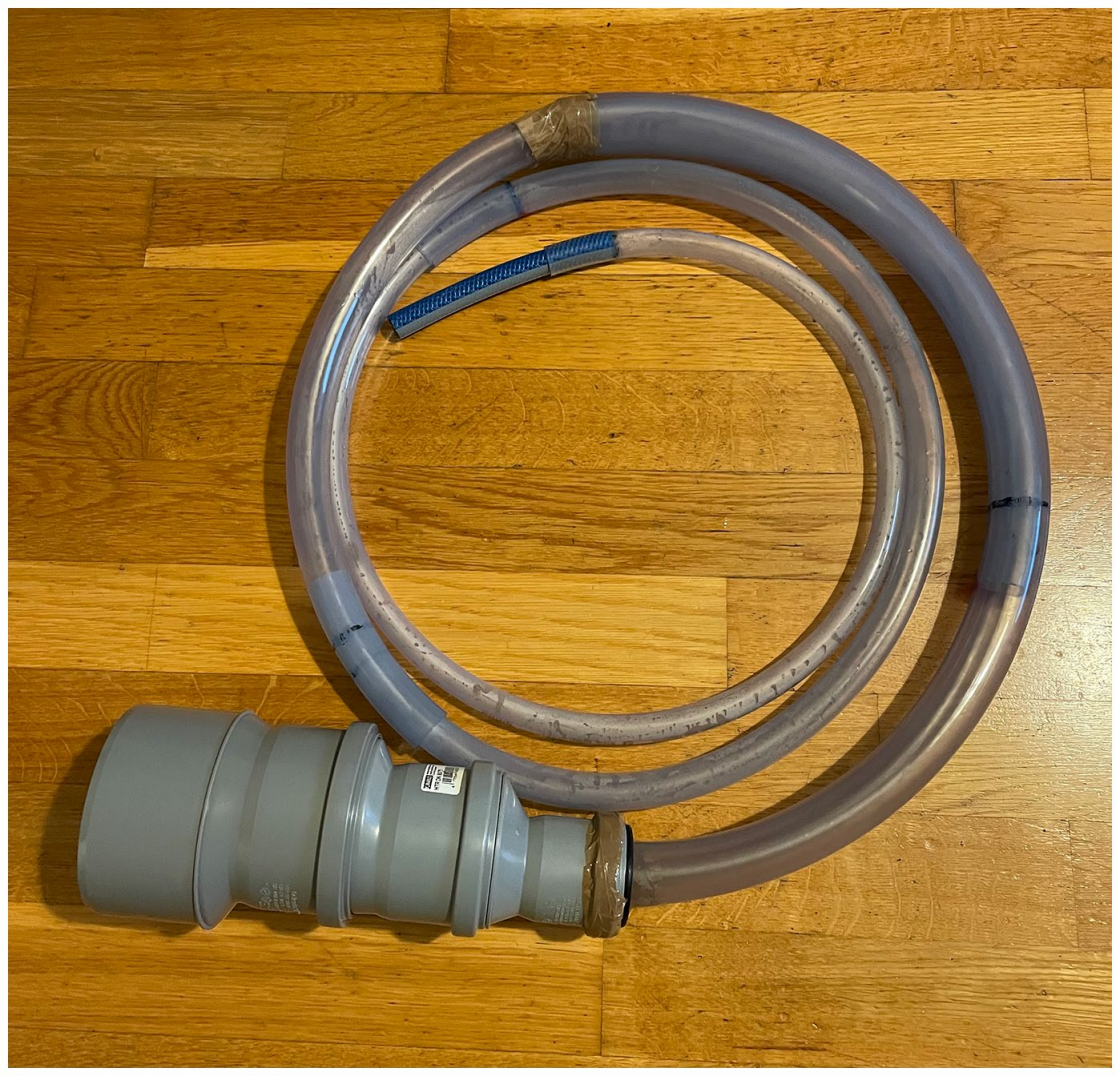
The MRI-compatible Tuba made of commercially available PVC tubes and polypropylene pipes.

Due to the size and ferromagnetic properties of a commercial tuba, it is not possible to perform MRI studies with a conventional instrument. To provide realistic playing conditions as much as possible, an MRI-compatible tuba was developed (see Figure 1) as previously accomplished for horn and trumpet experiments. Here, commercially available PVC tubes of increasing diameter were connected. At the end of the instrument, a bell made of polypropylene pipes was attached to provide the most realistic blowing resistance and to improve the sound quality. The instrument simulates a tuba without valves and is tuned in F. As a mouthpiece, all subjects used a plastic Helleberg Tuba mouthpiece.

#### Exercises

**Figure 2:**
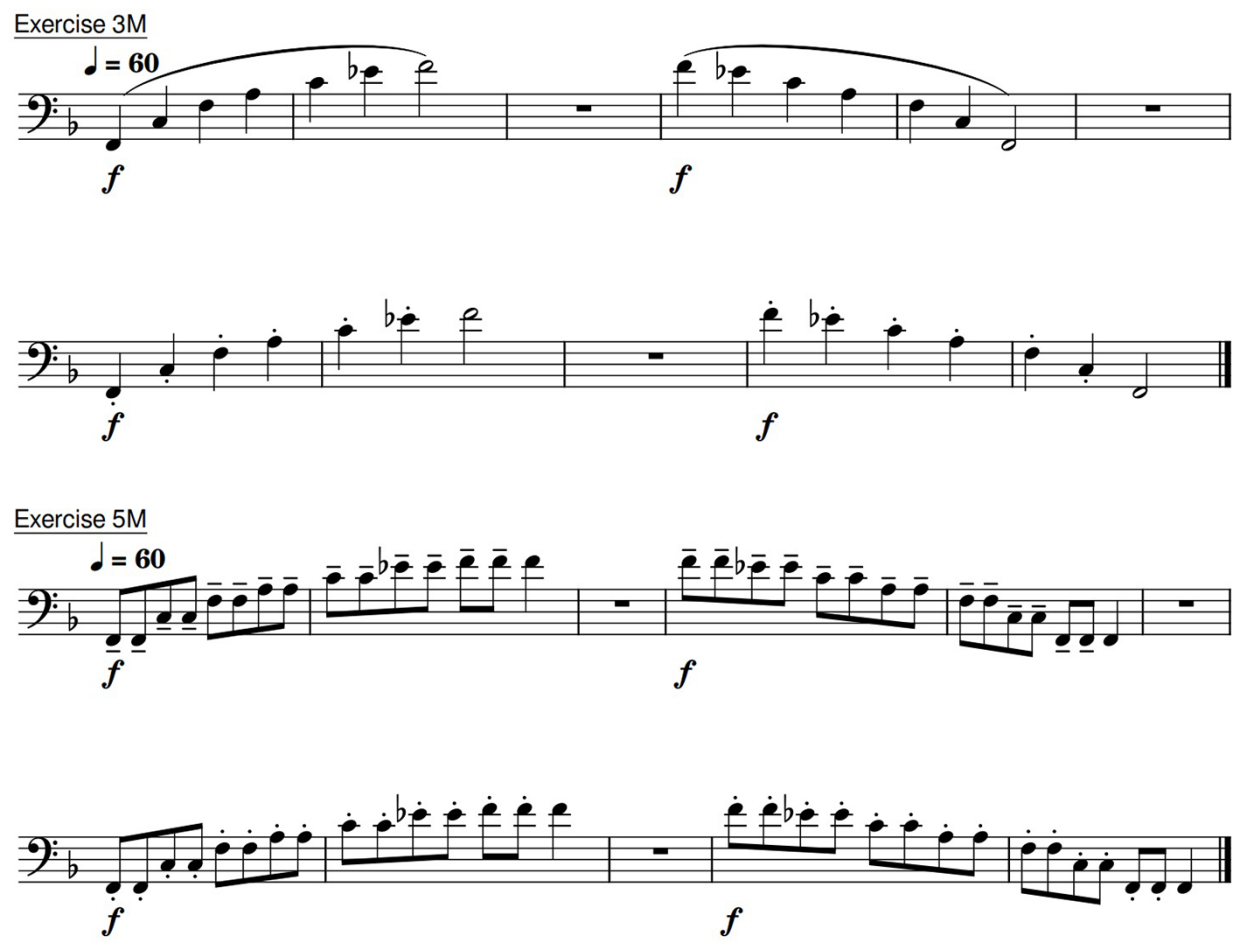
The exercises 3M and 5M as a foundation for the following analyses.

All subjects performed 2 different exercises designed to require particular technical skills that are fundamental in tuba playing (see Figure 2). The subjects were all very familiar with exercises like these. Exercise 3M involves ascending and descending harmonics, which were performed using a slurred and staccato articulation technique. Exercise 5M examines repeated articulations of each note in the same harmonic series using two different types of tongue articulation (tenuto vs. staccato). The tongued exercises were incorporated to highlight the problems of the dystonia patient who suffers from tongue articulation dysfunction. The exercises were sent to the subjects 1 week before the MRI measurement, and all subjects had 5–10 min directly before being studied in the MRI scanner to familiarize themselves with the MRI-compatible tuba.

Subjects played in a supine position in the scanner. They could see the exercises on a screen using a mirror. A 2-way communication intercom allowed contact between the experimental supervisor and the subjects. A microphone (Dual Channel-FOMRI, Optoacoustics, Or Yehuda, Israel) attached to the bell allowed the experimental supervisor to listen to and record the played exercise. The sound files were synchronized with the MRI videos as already performed in other studies (24). To achieve tempo consistency, the exercises were performed with an LED array as a visual metronome. If the performance of an exercise was not of professional quality in the opinion of either the experimental supervisor or the subject, the respective exercise was immediately repeated. This ensures that an adequate measurement of each exercise is available. Since the scanner noise during the measurements especially in the higher frequency range is relatively loud (about 90 dB at 1000 Hz), all subjects wore hearing protection as required by German law. However, the tuba sound played back in real-time was at lower frequencies (44–430 Hz) at about 95 dB. Since the sound was not masked, all subjects could hear their playing and perceive the sound quality and therefore were able to perform the exercises in good quality. No one had to discontinue the study.

In the evaluation of the results, the exercises 3M and 5M are particularly important (see Figure 2). Exercise 3M involves ascending and descending harmonics, which were performed slurred and tongued. This exercise is the foundation of many exercises, reflecting a large amplitude and all subjects are familiar with the requirements. Additionally, the ascending part of exercise 5M was analyzed, in which two different types of tongue articulation (tenuto vs. staccato) were presented. The tongued exercises mainly highlight the problems of the dystonia patient who suffers from tongue articulation dysfunction.

#### Real-time MRI

All MRI studies were performed at a field strength of 3 Tesla (Magnetom Prisma fit, Siemens Healthcare, Erlangen, Germany) using a 64-channel head coil. Real-time MRI was based on highly undersampled, radially encoded gradient-echo sequences (2.22 ms repetition time, 1.33 ms echo time, 5 degree flip angle, 9 spokes per image) in conjunction with serial image reconstruction by nonlinear inversion with temporal regularization (25). Accordingly, single-slice studies in a mid-sagittal position yielded a temporal resolution of 20 ms (i.e., the image acquisition time) corresponding to an MRI movie at 50 frames per second, while simultaneous (i.e., frame-interleaved) dual-slice acquisitions in a mid-sagittal and coronal orientation were obtained at 2 × 20 ms resulting in two MRI movies at 25 frames per second. All images had an in-plane resolution of 1.4 mm and a slice thickness of 8 mm covering a field-of-view of 192 × 192 mm^2^ with a resolution of 136 × 136 pixels. The spatiotemporal accuracy of these methods was experimentally assessed (26) and allows detection of small object movements with velocities of up to 25 cm s^−1^.

#### Data analysis

For the analysis of the data, a previously described and established analysis method using the computer software MATLAB (MATLAB R2015b, including ‘Image Processing Toolbox’, ‘Signal Processing Toolbox’ and ‘Statistics and Machine Learning Toolbox’) was consulted (7–9, 27). The software enables defined profile lines (PLs) to be superimposed over the images as an evaluation method.

**Figure 3:**
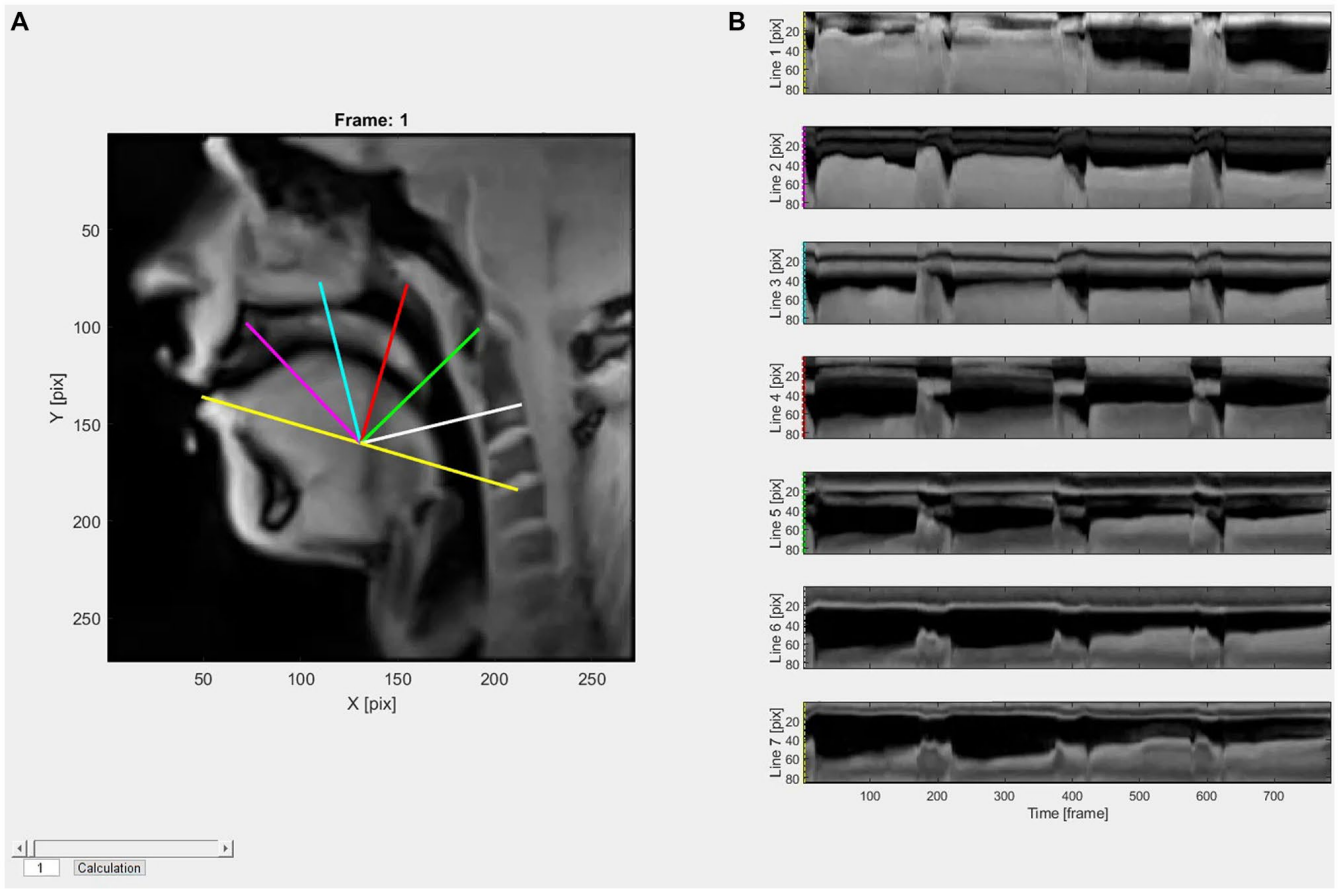
The localization of the 7 PLs is shown in panel **A**. The different PLs are visualized in different colors and are projected over the MRI images with the software MATLAB. Time-dependent changes in tongue edge position (pixels) along each line are presented in panel **B**. The colors at the beginning of each graph in panel **B** are linked to the PL colors in panel **A**.

Figure 3 (panel A) shows the locations of the seven PLs. Anatomical fixpoints used were the tip of the maxillary incisors for PL 1 and the upper edge of the C3 vertebral body for PL 7. The software then creates 7 PLs separated by angles of 30°, which can be used to infer changes in oral cavity space (OCS) by quantify the localization of the tongue edge. Downward movements of the tongue reflect increase in OCS and upward movements reflect decreases in OCS. As already described, the tongue movements are indicated by changes in the position of the tongue edge along each line over time (see Figure 2, panel B). These positional changes can be quantified and can be used to compare tongue movement patterns among different groups (27). The length of the PLs is 80 pixels, where position 0 is at the single, outer end of the line. The maximum value 80 is reached at the origin position, where all lines converge. Thus, a larger value represents a lower tongue position, and consequently, a large OCS above the tongue. Conversely, the value becomes smaller as the tongue moves upwards, indicating a smaller OCS. PLs 2, 4 and 6, which are representative of the anterior, intermediary, and posterior OC, respectively, were selected for the evaluation of the study results.

For both exercises, the start and end time was determined for each tone using the audio software, Audacity®. With these times, MATLAB was able to calculate a mean, minimum, maximum, and standard deviation for the tongue edge pixel position (PP) during the sounding of each note. The number of frames analyzed ranged from a minimum of 7 frames for particularly short tones (exercise 5M staccato) to a maximum of about 65 frames for slurred 7-note harmonics (exercise 3M slurred).

To illustrate differences between healthy and dystonic subjects and integrate the data available an additional explorative Bayesian statistical modeling was made. For the analyses, participants were divided into two groups, namely healthy (*N* = 11) and dystonic (*N* = 1) *groups*. Moreover, the seven *notes* composing the exercise 3M were treated as ordered variable and progressively coded with integer numbers from the lower to the higher note. A Bayesian mixed effects regression model was run to measure changes in tongue position during the experiment and identify movement patterns referable to EmD across the three PLs considered. The model entered tongue positions data measured in PP as criterion and *note, group* and the interaction between the two covariates as fixed effects. Moreover, it included random intercept per *participant*, random intercepts per *PL* nested on *participant* as well as random slopes per *note* as random effects. To increase the robustness of the results, the response variable was modeled as a student-t probability distribution. In the present manuscript, effect estimates are reported together with 95% Credibility Intervals (CI).

The analyses were performed in RStudio (28), through the R-package brms (29).

## Results

### Healthy musicians

**Figure 4:**
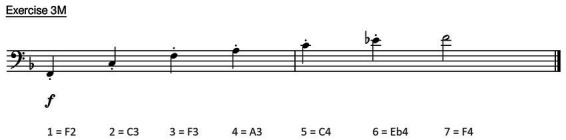
Exercise 3M, a 7-note harmonic series in ascending and tongued form.

**Figure 5:**
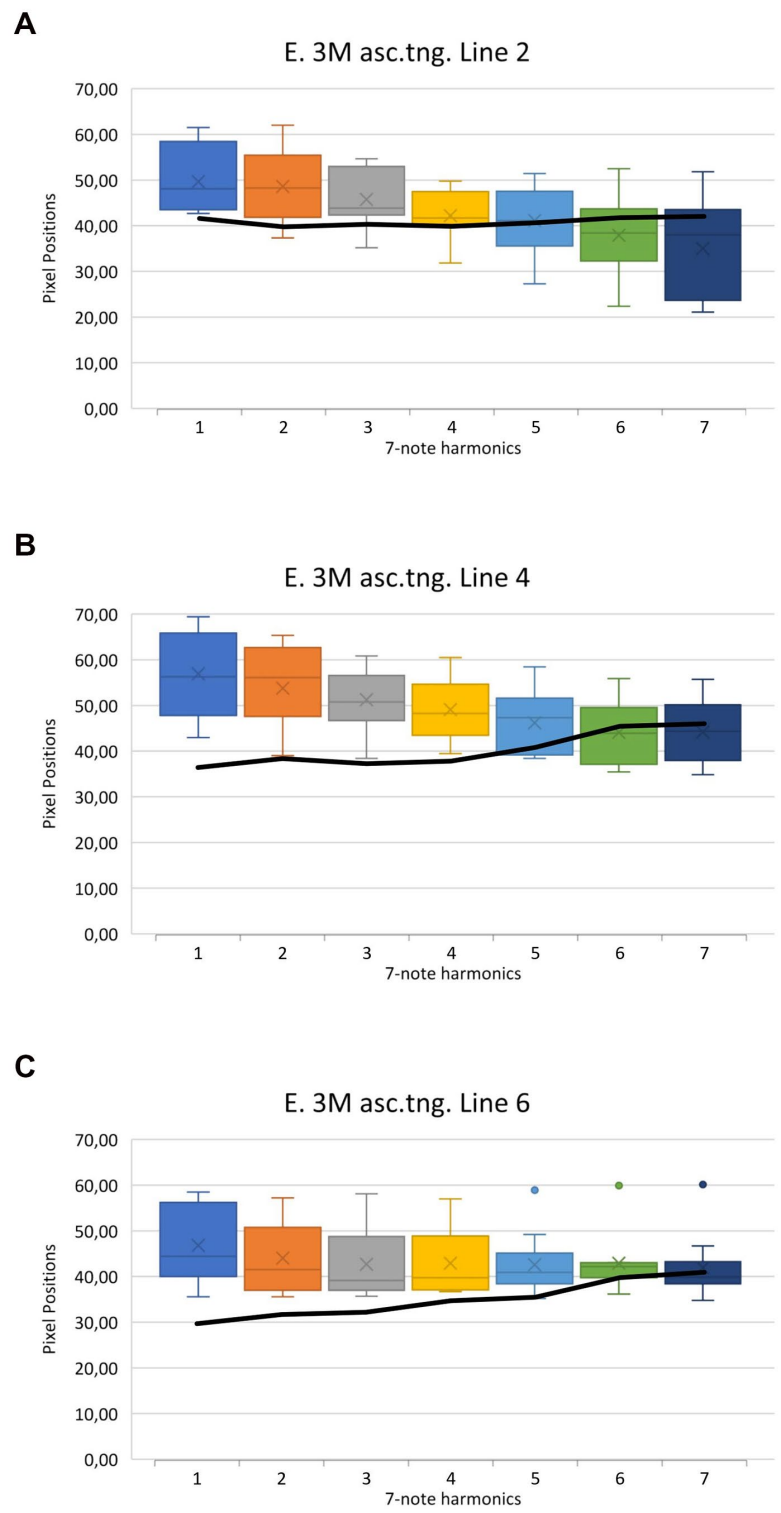
Results of exercise 3M in ascending and tongued form (see Figure 4). Panel **A** represents the tongue edge position along PL 2, panel **B** PL 4, and panel **C** PL 6 corresponding to the anterior, intermediate, and posterior oral cavity, respectively. The data of the healthy tuba players are shown in the boxplots. The black line reflects the data of the dystonia patient.

The results of exercise 3M (see Figure 4), in ascending, staccato tongued playing style are shown in Figure 5. The data of the healthy tubists are presented in the boxplots. Each boxplot contains the data from 11 healthy tubists and displays the mean (x), median (horizontal line within each box), interquartile range (box), and minimum and maximum data points at the respective ends of the whisker. The black line in each panel represents the mean position of the tongue edge in the dystonic subject during each note. The seven boxplots represent the seven played notes of exercise 3M. So boxplot 1 quantifies the lowest note (F2) and boxplot 7 the highest note (F4) of this exercise.

Several observations concerning tongue movements in the healthy group were made. The greatest change occurs in PL 2 (see Figure 5, panel A, magenta PL in Figure 3), in the anterior region of the OC. Comparing the mean value of the lowest tone (PP = 49.62) and that of the highest tone (PP = 35.03), an apparent reduction of the OCS in the anterior region by 14.59 pixels can be observed. This corresponds to a reduction of 29.4%. At the intermediate part of the tongue, in PL 4 (see Figure 5, panel B, red PL in Figure 3), a continuous decrease of the OCS can be inferred as well. The value decreases from the lowest tone with PP 56.94 to PP 44.17 at the highest tone. This is equivalent to a reduction of 12.77 pixels and 22.4%. In the posterior region of the oral cavity, at the root of the tongue, considerably less change can be observed (see Figure 5, panel C, white PL in Figure 3). While there is still a perceptible reduction in the lower tones, the higher the tone, the smaller the reduction. Calculations show a decrease from PP 46.83 to PP 41.93 from the lowest to the highest note. This suggests a reduction of the OCS by 4.90 pixels, which corresponds to a 10.5% decrease.

Furthermore, Supplementary Video 1, depicting exercise 3M, clearly demonstrates that in a representative example of a healthy tubist a change occurs mainly in the anterior part of the oral cavity, while the pharyngeal aperture in the posterior part of the oral cavity tends to remain constantly open. The entire results of Exercise 3M and Exercise 5M ascending are displayed in the Supplementary Data Sheets 1, 2. They show the same effects as in the described exercise 3M and underline the above-mentioned observations.

### Patient with EmD

The case report of the patient with EmD is also shown in Figure 5. The black line represents the mean value of each tone of the patient in exercise 3M in ascending, staccato tongued form (see Figure 4).

In the anterior OC, represented by PL 2, only minimal changes can be observed (see Figure 5, panel A). Comparing the mean value of the lowest tone (PP = 41.90) with that of the highest tone (PP = 42.36), an apparent increase in size of the OCS, as suggested by a downward movement of the tongue edge by 0.45 pixels (1.1%), can be inferred. In PL 4, a greater increase in OCS is suggested (see Figure 5, panel B). While in healthy tubists there is a suggested reduction of the OCS, in this intermediate region, the opposite is seen for the dystonic patient. The tongue edge descends from PP 36.59 to PP 46.15. This 9.56 pixels (26.1%) descent suggests an increase in OCS for the patient.

The greatest increase in OCS can be observed in the patient’s posterior oral cavity, represented by PL 6 (see Figure 5, panel C). Here the tongue edge descends from PP 29.85 at the lowest tone, by 11.26 pixels to a PP of 41.11. This corresponds to a 37.7% lowering of the tongue edge.

It is noteworthy that there is hardly any movement in the anterior region of the OC. The middle and posterior regions of the tongue make larger movements, which are opposite to those of healthy tubists. The movements of the dystonia patient can be seen in Supplementary Video 2, depicting exercise 3M.

Nevertheless, due to the reduced statistical power, these visible changes in OCS were not significantly different between healthy subjects and the dystonia patient (*p* > 0.05).

The observations are supported by the results of the explorative statistical analysis and Bayesian mixed effects regression model performed in the study. As shown in Table 2, at the beginning of exercise 3M healthy tubists showed a mean tongue position value of PP 50.75 while this value was PP -15.84 lower for the EmD patient. Moreover, at each progressive *note* included in the exercise the tongue was lowered on average by PP −1.83 in the healthy subjects while it was moved to a higher position in case of the EmD tubist, by PP 1.25 per *note* (PP 3.08 – PP 1.83 = PP 1.25).

**Table 2:** Bayesian mixed effects regression models measuring changes in tongue position during the experiment and movement patterns referable to EmD.

Fixed effects	Estimate	Est. error	CI lower	CI upper
Intercept	50.75	2.00	46.73	54.68
Note	−1.83	0.39	−2.60	−1.06
EmD_group	−15.84	7.02	−29.27	−2.31
Note: EmD_group	3.08	1.34	0.43	5.73
Random effects				
~Participant				
sd (Intercept)	5.03	0.88	3.35	6.76
sd (note)	0.88	0.34	0.18	1.57
cor (Intercept, note)	−0.25	0.32	−0.77	0.45
~Participant/Line				
sd (Intercept)	7.44	0.45	6.57	8.34
sd (note)	1.47	0.18	1.15	1.84
cor (Intercept, note)	−0.59	0.13	−0.79	−0.29
sigma	1.81	0.25	1.34	2.30
nu	6.38	5.05	2.18	20.99
Coefficients of determination				
Conditional *R*^2^	0.93	0.01	0.92	0.94
Marginal *R*^2^	0.23	0.07	0.10	0.38

The results take healthy tubist (*N* = 11) as a reference. EmD_group = tubist affected by embouchure dystonia (*N* = 1).

### Effects of different articulations in healthy tubists

Furthermore, we analyzed the difference of various playing techniques.

**Figure 6:**
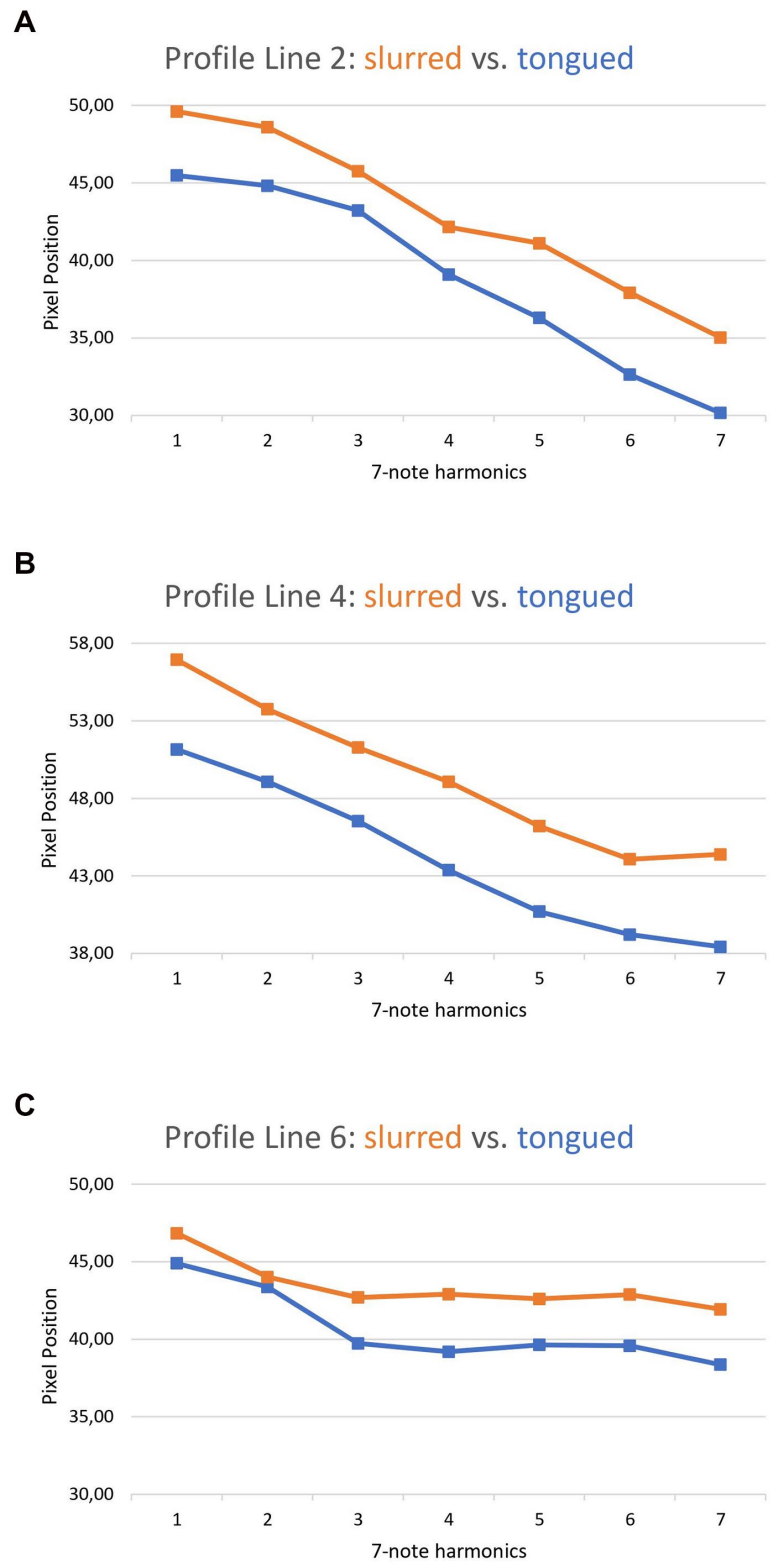
Comparison between the slurred and tongued notes of exercise 3M. **A**: profile line 2, **B**: profile line 4, **C**: profile line 6.

In addition to the change in pitch (see Figure 5) the measurement results of slurred and tongued notes were compared. Figure 6 presents the analysis of exercise 3M, comparing the slurred and the staccato tongued version (see Figure 2, first line vs. second line). The orange line represents the means of the slurred harmonic series. The blue line summarizes the data of the staccato tongued notes of the same harmonic series. It was found that slurred notes were always associated with a larger OCS than tongued notes.

**Figure 7:**
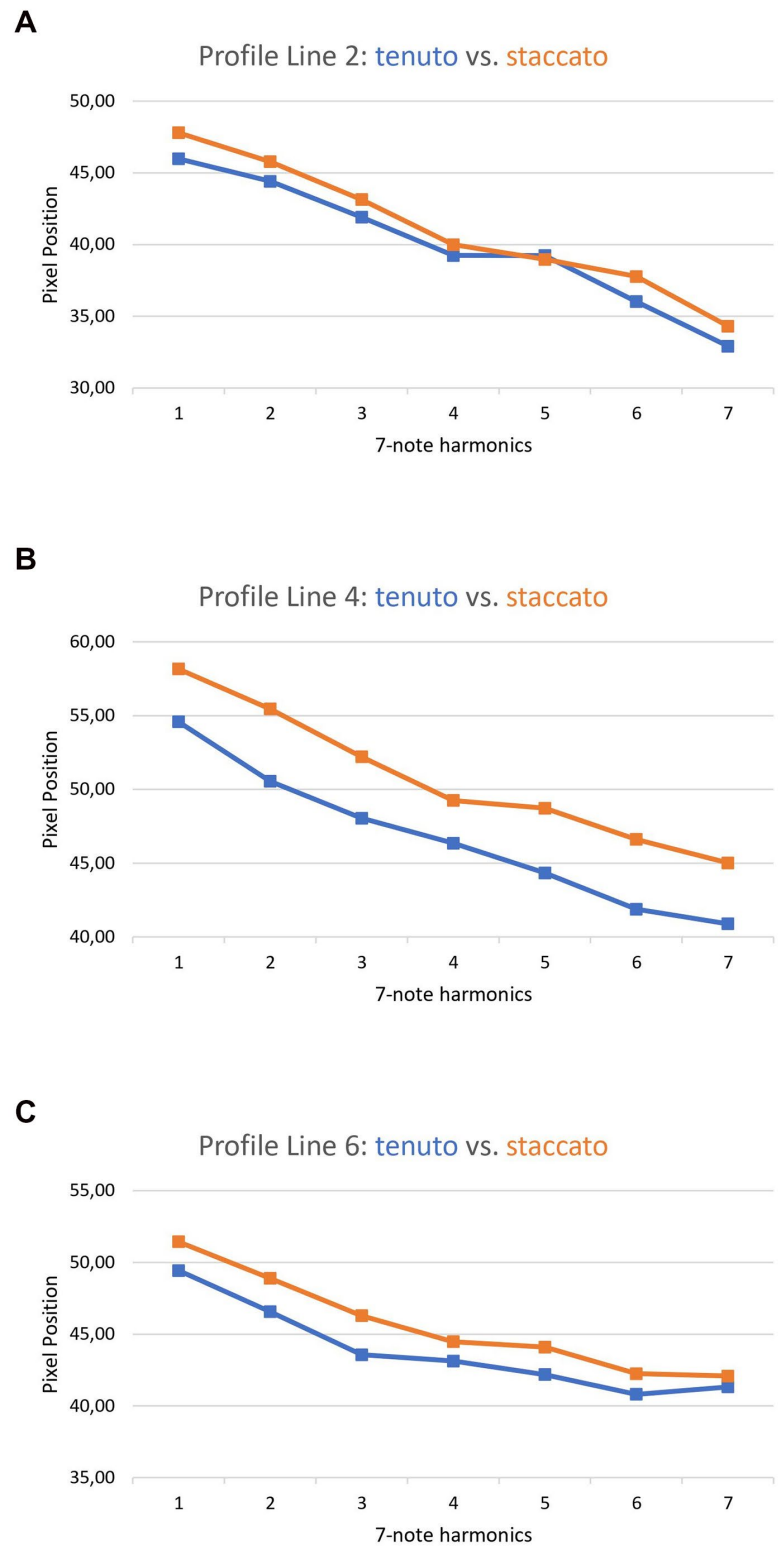
Comparison between the tenuto and the staccato played notes of exercise 5M. **A**: profile line 2, **B**: profile line 4, **C**: profile line 6.

When comparing the tenuto and staccato playing techniques in the ascending harmonics of exercise 5M (see Figure 2, third line vs. fourth line), a larger OCS was shown in all PLs during staccato playing. This is depicted in Figure 7. The orange line describes the mean values of the staccato tongued notes of ascending exercise 5M. The blue line reflects the tenuto tongued notes of the ascending exercise 5M.

## Discussion

### Physiology of brass playing

This study is the first to quantitatively describe the tongue movements of professional tuba players during playing. It was observed that the largest tongue movements occur in the anterior part of the oral cavity. The further back the measurements were taken, the smaller was the correlation between tongue movement and pitch change (see Figure 5). The common feature of the movements of the tongue in the regions of the oral cavity studied is a reduction in the size of the OCS when the frequency is higher and an increase in the OCS the lower the pitch played.

Iltis et al. (7) demonstrated already in the first pilot study with real-time MRI differences in OCS depending on whether low or higher pitches had to be played. They compared four individual players of trumpet, french horn, trombone and tuba and found in all four brass instrumentalists more movements in the anterior part of the tongue, suggesting that this part of the tongue was used at a valve, regulating air flow and airspeed. Already in this pilot-study, characteristic differences were also demonstrated, showing that the tuba player moved his tongue far less in ascending slurs as compared to the trumpet player and the French horn player. However, in this experiment a mouthpiece connected to a buzzing resistance device (BERP ®) and no real instrument was used, a completely different situation, since rapid adaptation of air guidance to different pitches and to intonation and sound quality was not necessary.

In the current study, these preliminary results were probed in a larger number of healthy players, using a real instrument, and applying different playing techniques such as staccato and legato.

The acquired data enables a comparison with previously published studies on tongue movements in professional musicians.

Generally, in all instrument groups studied the anterior part of the oral cavity becomes smaller with higher pitches. There are, though, characteristic differences: in trumpeters, Hellwig et al. (9) observed that with increasing pitch, there was a particularly strong reduction of the diameter of the air channel in the anterior part of the oral cavity (by 70.9%), while the intermediate part remained constant, and the posterior part showed an increase in the oral cavity of 37.1%. A strong lifting of the tongue was also observed in professional horn players, especially in the anterior third of the oral cavity (8). These effects were attributed to the different physics of sound production, requiring higher intra-oral pressures and speed of airflow in trumpeters and horn-players in the high register as compared to tuba-players (30). We assume that high brass instrumentalists in the higher registers rely more on Hagen-Poiseuille’s streaming laws by extremely narrowing the air channel in the anterior part of the oral cavity thereby using tongue and hard palate to form and rapidly adapt a valve-like structure. In the posterior part, e.g., the pharynx, similarly differences can be shown, such as an increase in the OCS in trumpet players and a decrease in tuba players. This is most probably related to the higher intraoral pressures in trumpet-players, pushing the root of the tongue forward. In the field of musicians’ medicine, it is known that these heightened pressures in the throat may occasionally cause laryngoceles or pharyngoceles, evaginations of the mucous membrane of the laryngeal and pharyngeal ventricles (31, 32). Compared to high brass instruments, it seems reasonable to assume that tuba players modulate and adapt the required lower air pressures and higher air volumes predominantly with the diaphragm and the abdominal musculature and to a lesser extent with the tongue. To prove this hypothesis, however, simultaneous recording of intraoral air pressures, sound pressure levels, and real time MRT in low and high brass players is required.

When comparing playing techniques, it was noticed that a slurred or a staccato playing style is accompanied by an increase in OCS in all regions of the oral cavity that were examined (see Figures 6, 7). This shows the importance of the whole tongue for certain playing techniques. Using these data, the corresponding tongue movement patterns can be assigned to specific playing techniques. This application is already used as a teaching method by playing an exercise first slurred, then tongued, while attempting to maintain the large OCS associated with the slurred version also in the tongued version, because a larger OCS is associated with better sound quality.

### Comparison of the dystonia patient with healthy players

When the tongue movements of the dystonia patient are contrasted with those of the reference group, it is noticeable that hardly any muscle movements are measurable in the anterior third of the affected player’s tongue. While most of the changes in the healthy players could be detected in this area, the dystonia patient demonstrated a constant position of the tongue apex across all ascending notes (see Figure 5, panel A). Whereas healthy tubists separate each note from the next with a movement of the apex of the tongue (see Supplementary Video 1), these movements are not present in the dystonia patient (see video 2). This lack of flexibility in the anterior third of the tongue did not only affect the adjustments to higher pitches, but also the push movements in tongued articulations. Here, the patient’s symptoms of “difficulties in tonguing” becomes obvious. To compensate for the lack of control of the tongue he produces rapid contractions of the diaphragm, thus producing the articulation “ha” instead of “da.” Furthermore, it is noteworthy that in the middle and posterior sections of the tongue, also differing motor strategies can be described (see Figure 5, panels B and C). While the healthy subjects produce a reduction of the OCS by the tongue, the patient shows an enlargement in PL 4 and 6. These movement patterns correspond to an inefficient technique, which may be associated with increased muscle tension of the embouchure and non-task-specific muscles, as previously described (8). This probably compensates for the impaired movement patterns allowing the patient to play the exercise sounding almost correctly. Indeed, when comparing the sound recordings of the patient with the healthy subject in Supplementary Videos 1, 2, there is little difference in stability of the fundamental frequency, which is mostly severely affected in EmD (33). However, the patient struggles with his slow and reduced tongue attack, leading to a “soft” sounding articulation. Furthermore, in the ascending slurred exercise lack of flexibility of the tongue leads to missing the highest pitch in the first attempt.

Comparing the dystonic symptoms of the tuba player to other brass instrumentalists yields interesting communalities and differences. Both, our tuba player and horn players suffering from EmD tend to create a smaller OCS in the three regions studied as compared to healthy tuba or horn-players (8). Furthermore in keeping with the result of the tuba player, horn-players suffering from dystonia show a tendency to decrease the OCS on ascending notes, however healthy horn players present a far wider range of tongue movements (8). Such a “stiffening” and lack of flexibility of tongue movements seems to be a common trait of EmD patients. In EmD trumpet players, the size of the OCS was more variable during sustained notes and in one patient the tongue showed a very unsteady pattern with wave-like movements [see Supplementary Video 2 in (9)].

### Pathophysiology of EmD and consequences for brass-player pedagogy

The major limitation of this study is that it only involved one EmD patient making generalizations to other affected tuba players difficult. We were unable to recruit more patients, since EmD in tuba players is extremely rare. In the last 28 years, out of a total of more than 1,500 dystonia patients, only 7 were professional tuba players and only three were younger than 40 years. From these, only one was willing to participate in this study. It is an intriguing question, why EmD is so infrequent in tuba-players. As mentioned in the introduction, the origin of EmD is multifactorial: on the bases of a genetic predisposition (34) and inadequate stress-coping strategies (18, 35) several triggering factors contribute to the disorder. Most important are virtuosic, fast, repetitive, highly controlled and controllable movement patterns (14). Contrasting to trumpet or french horn playing, this is not frequently required in tuba literature. Furthermore, muscular strain or overuse is contributing to origin and onset of the disease (36). Amongst all brass instrumentalists, tuba players have the lowest risk of the “loss-of lip” syndrome, muscular fatigue, and overuse of embouchure (2).

Concerning the pathophysiology of EmD, it is now generally acknowledged, that it is a network disorder including cortical brain areas but also basal-ganglia and cerebellar loops (37). These dysfunctional brain networks lead to a lack of inhibition of motor outputs, and thus compromise the rapid adaption of motor activations contributing to skilled movements of embouchure muscles and/or the tongue (38). The observed “stiffness” of the anterior part of the tongue of the EmD patient is probably one of the consequences of lack of inhibition. This in turn could lead to compensatory movements of the oral cavity and embouchure muscles, which may dominate the clinically visible dysfunctional movement patterns. However, a word of caution, the current study provides evidence of a case where a divergent motor strategy may be consequent of or contributing to the disorder. Since all cases of musicians with EmD are unique, there is a clear need for additional studies.

The embouchure of brass players as a very complex sound production and sound changing unit should be further investigated and compared in future studies to enhance understanding of efficient playing techniques that might be effective in the prevention of EmD. To understand the nature and mechanisms more completely, it would be interesting to include measurements of airflow, diaphragm movements, pressure in the oral cavity, and the tension of individual muscles as additional parameters in future studies.

For dystonia patients with individual tongue movement problems, individual exercises using certain playing techniques could be created to induce specific movement patterns. Ultimately, this could lead to a scientifically based “retraining” as it has been developed for hand dystonia (39).

## Conclusion

Using real-time MRI imaging, healthy professional tubists and a professional tuba player suffering from EmD were studied regarding tongue movement patterns in the oral cavity.

The analysis of the MRI videos shows that during performance of an ascending harmonic sequence in healthy tubists, there is a decrease in OCS across the entire oral cavity, with the anterior region showing the most reduction, and the posterior region tending to exhibit more attenuated changes. This effect is, however, less pronounced as in high brass instruments relying on higher air pressures in the upper register. In terms of playing technique, we assume, that tuba players modulate and adapt air pressure required for sound production with the diaphragm and the abdominal musculature, whereas high brass instruments rely more on narrowing the air-channel using tongue and hard palate to form and adapt it.

The fact that the tongue movements of the EmD patient deviate strongly from the healthy movement patterns suggest that movement disturbances in a small area of the tongue may affect the entire embouchure, leading to maladaptive adjustments that effect tuba performance. To better identify the compensatory movements, future studies should include not only tongue and embouchure movements, but also other parameters such as diaphragmatic movements, muscle tension or airflow with a greater number of dystonia patients.

## Data availability statement

The raw data supporting the conclusions of this article will be made available by the authors, without undue reservation.

## Ethics statement

The studies involving human participants were reviewed and approved by Ethics Committee of the Hannover Medical School: No 9914 Bo-S-2021. The patients/participants provided their written informed consent to participate in this study.

## Author contributions

RN designed the experiment, recruited the subjects, built the MRI compatible Tuba and performed the experiment, evaluated the data, and wrote the manuscript. PI designed the experiment, analyzed the data, and wrote the manuscript. DV designed the experiment, developed the real-time MRI-software and performed the measurements, and contributed to the manuscript. JF designed the experiment, developed the real-time MRI software, conducted the measurements, contributed to data evaluation, and contributed to the manuscript. EA designed the experiment, recruited the subjects, obtained funding, investigated the dystonia patient, attended the measurements, and contributed to writing the manuscript. EP discussed the results, calculated the statistics and contributed to the writing of the manuscript. All authors contributed to the article and approved the submitted version.

## Funding

This research was funded by Hannover University for Music Drama and Media Max Planck Institute for Interdisciplinary Sciences Göttingen.

## Conflict of interest

The authors declare that the research was conducted in the absence of any commercial or financial relationships that could be construed as a potential conflict of interest.

## Publisher’s note

All claims expressed in this article are solely those of the authors and do not necessarily represent those of their affiliated organizations, or those of the publisher, the editors and the reviewers. Any product that may be evaluated in this article, or claim that may be made by its manufacturer, is not guaranteed or endorsed by the publisher.

## Supplementary material

SUPPLEMENTARY VIDEO 1 Tongued ascending harmonics in a healthy Tuba player.

SUPPLEMENTARY VIDEO 2 Dystonic Tuba player: Ascending harmonics with diaphragm. Tongue movements are impaired and dysfunctional.

SUPPLEMENTARY DATA SHEET 1 Results of exercise 3M and 5M in ascending and descending slurred and tongued mode as well in staccato. Solid line: Dystonia Patient.

SUPPLEMENTARY DATA SHEET 2 Individual results of exercise 3M, Solid black line: dystonia patient.

## Abbreviations

EmD: Embouchure Dystonia
MRI: Magnetic Resonance Imaging
OCS: Oral Cavity Space
PP: Pixel Position
PL: Profile Line
