# Supplementary figures and images for: Movement patterns in tuba playing: comparison of an embouchure dystonia case with healthy professional tuba players using real-time MRI imaging
Source: Front Neurol. 2023 May 10;14:1106217. doi: 10.3389/fneur.2023.1106217 (PMC10206072; doi:10.3389/fneur.2023.1106217)

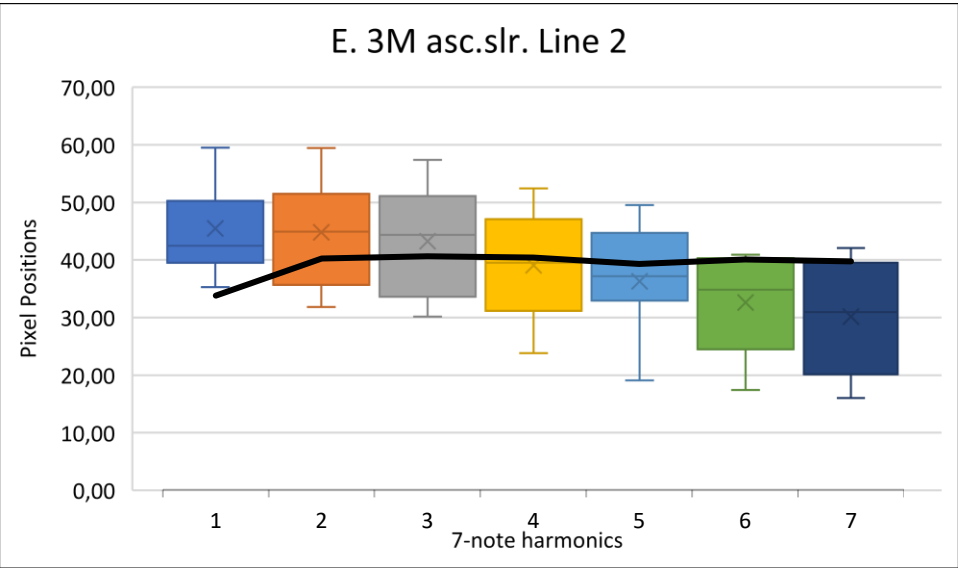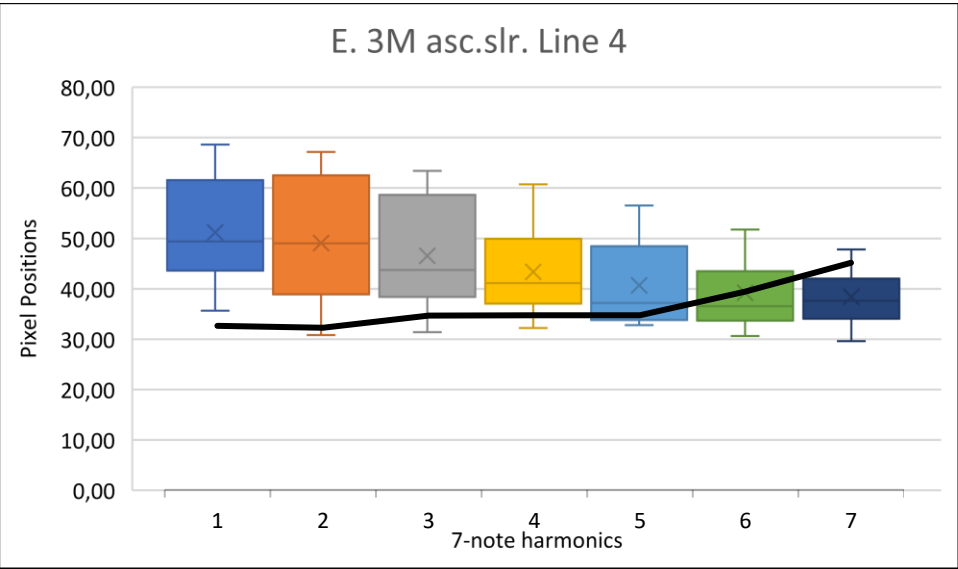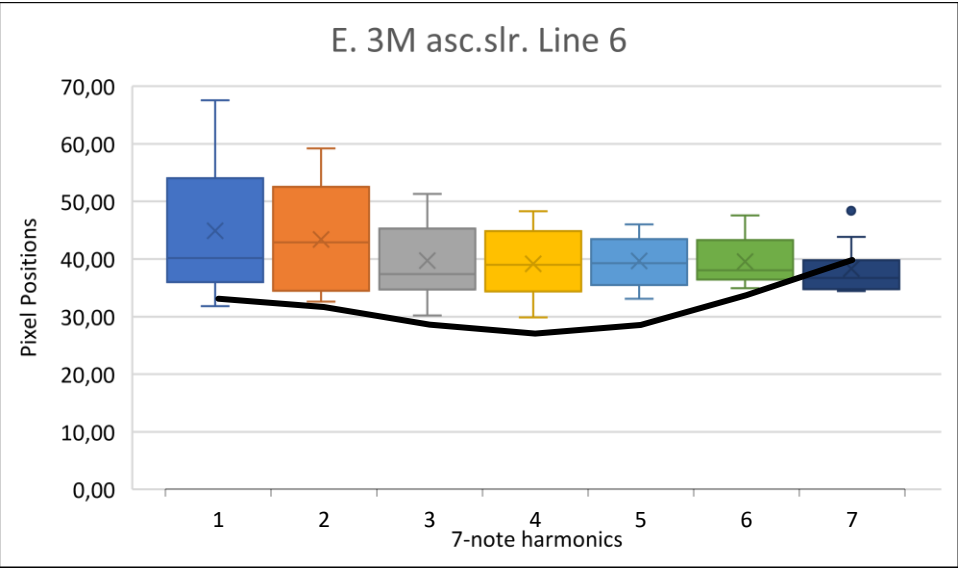

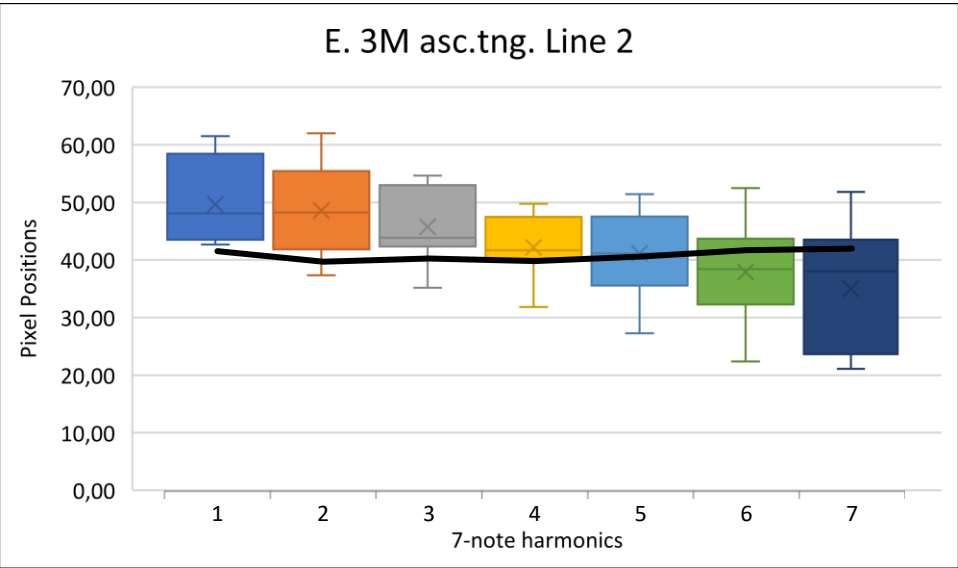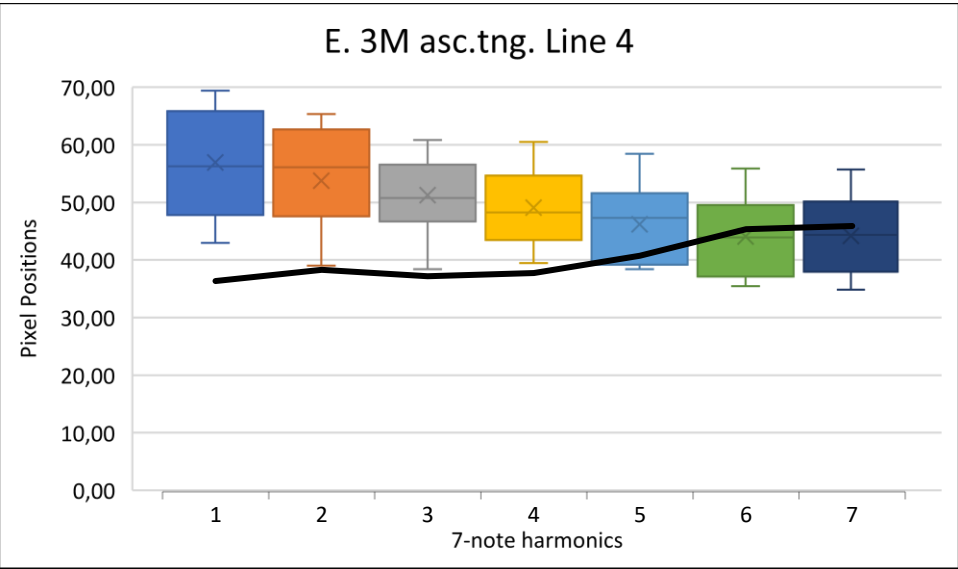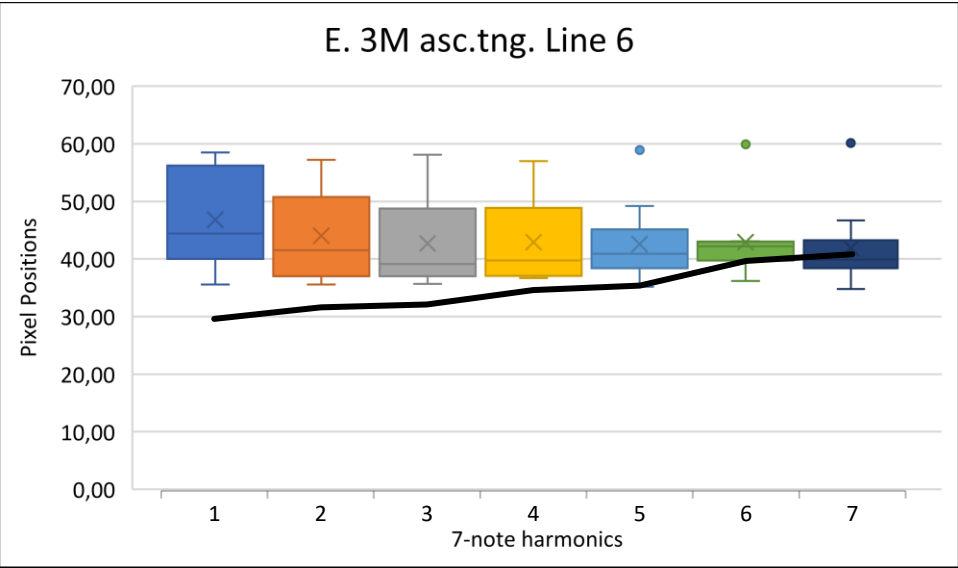

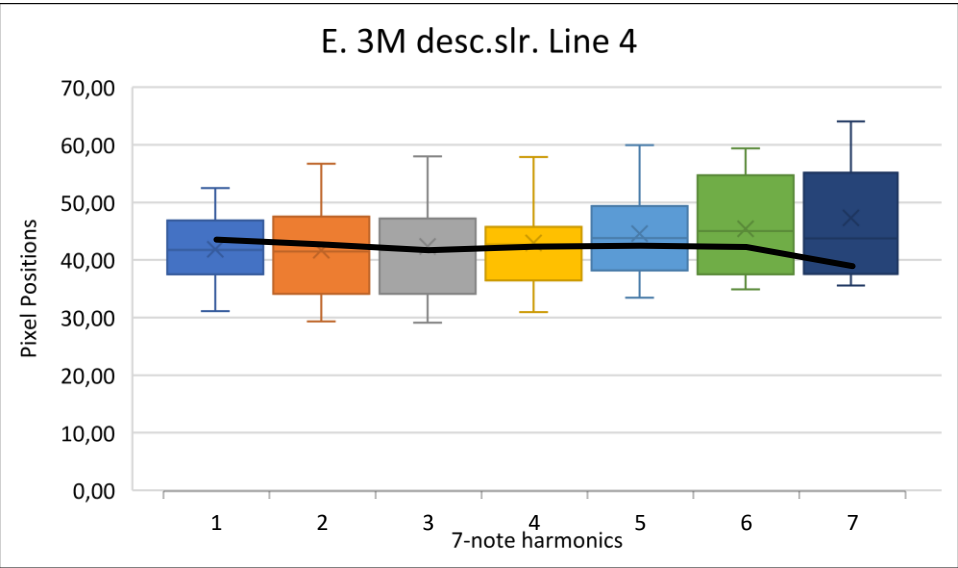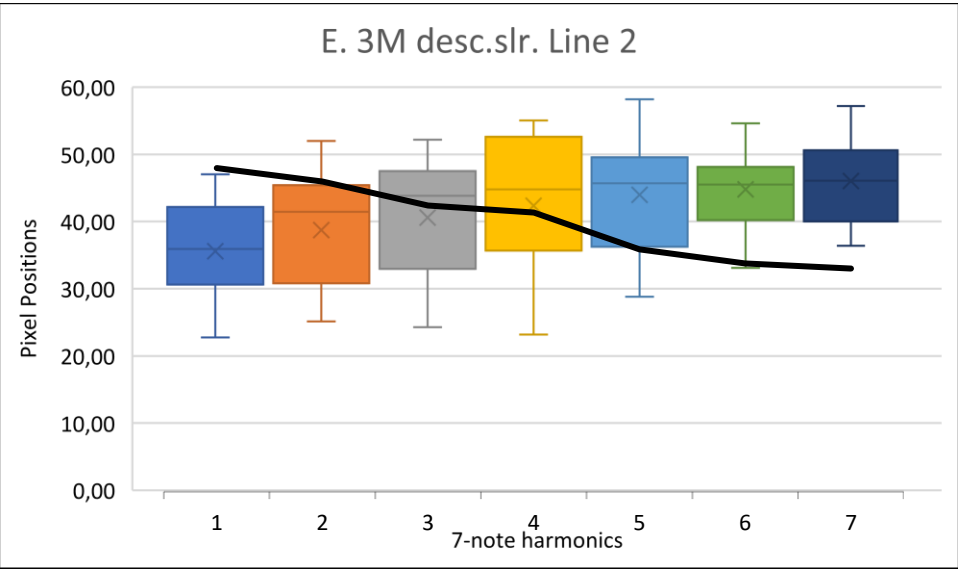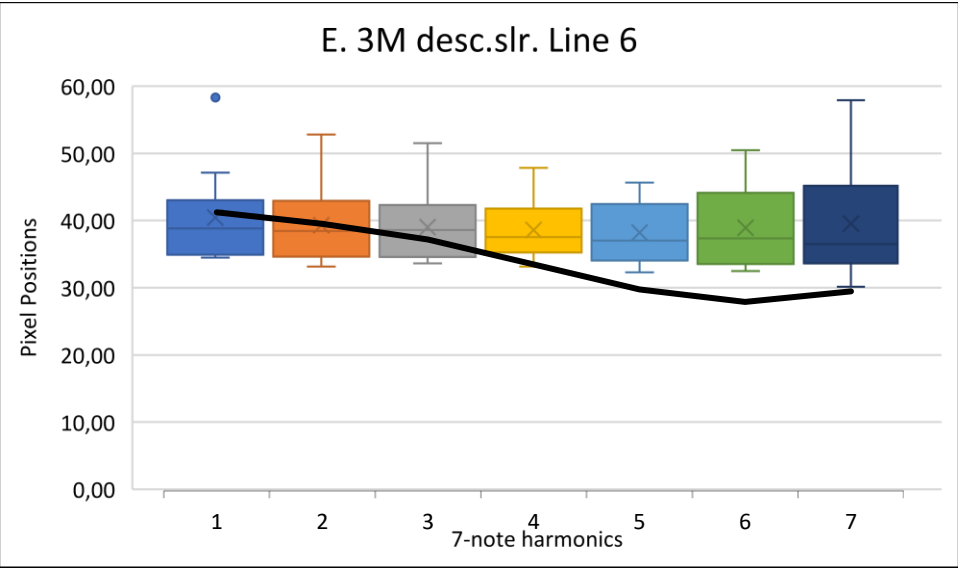

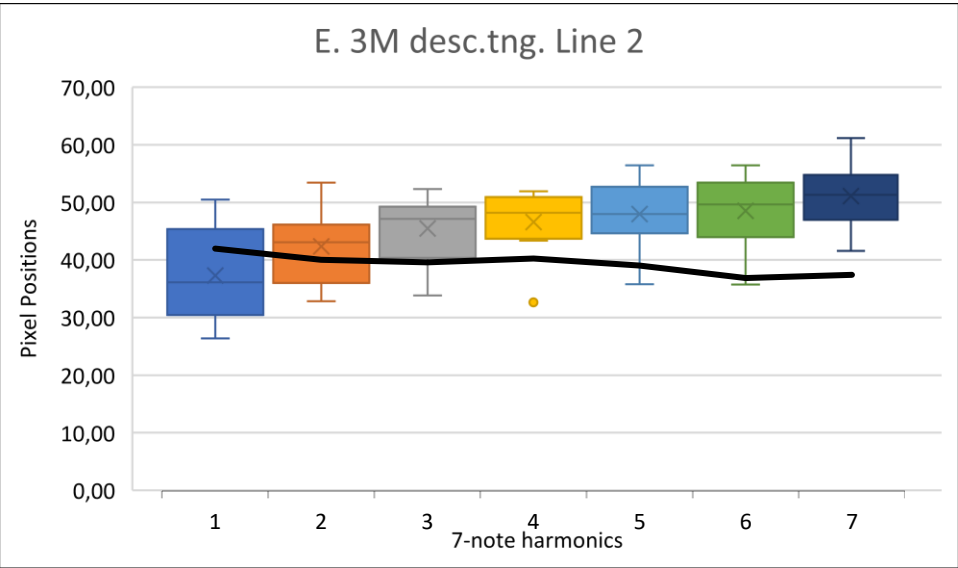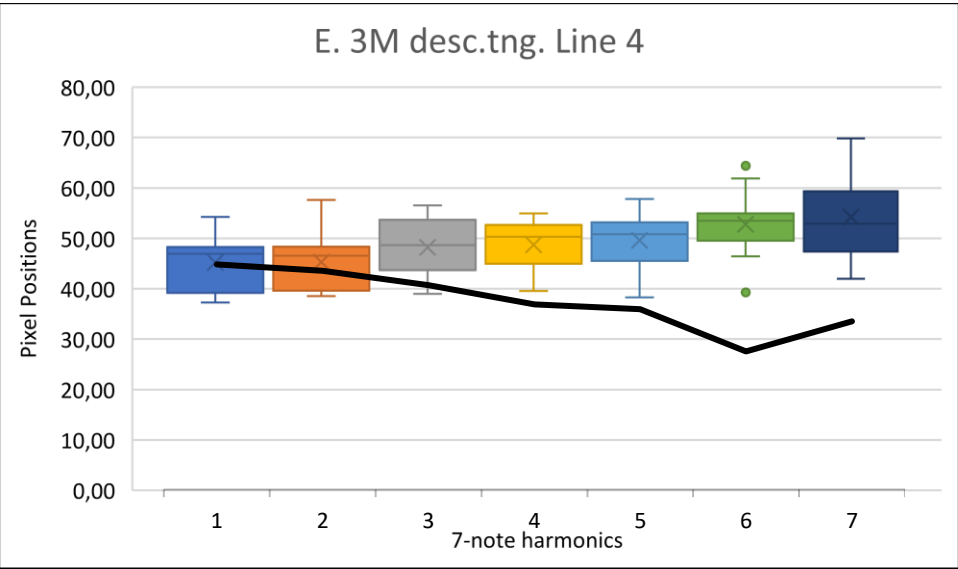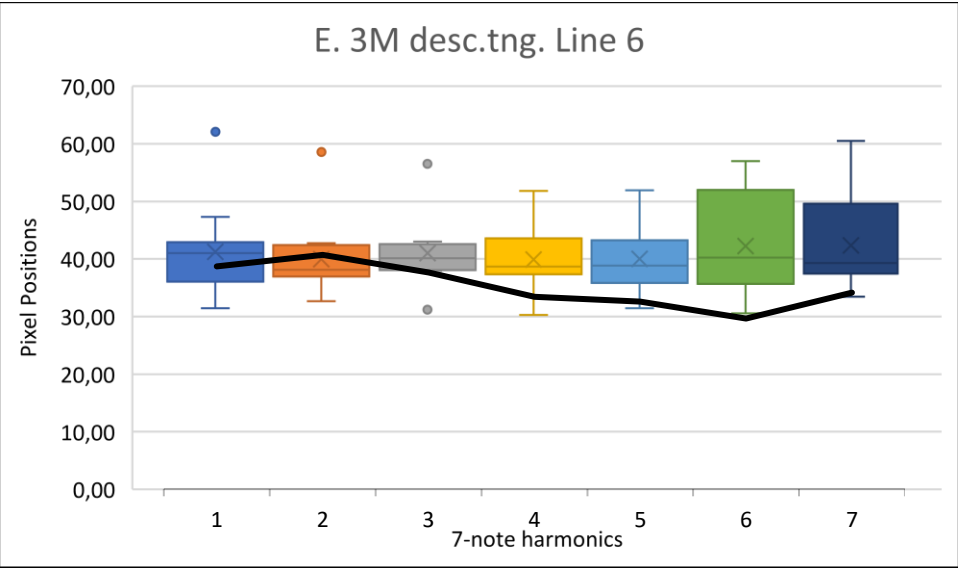

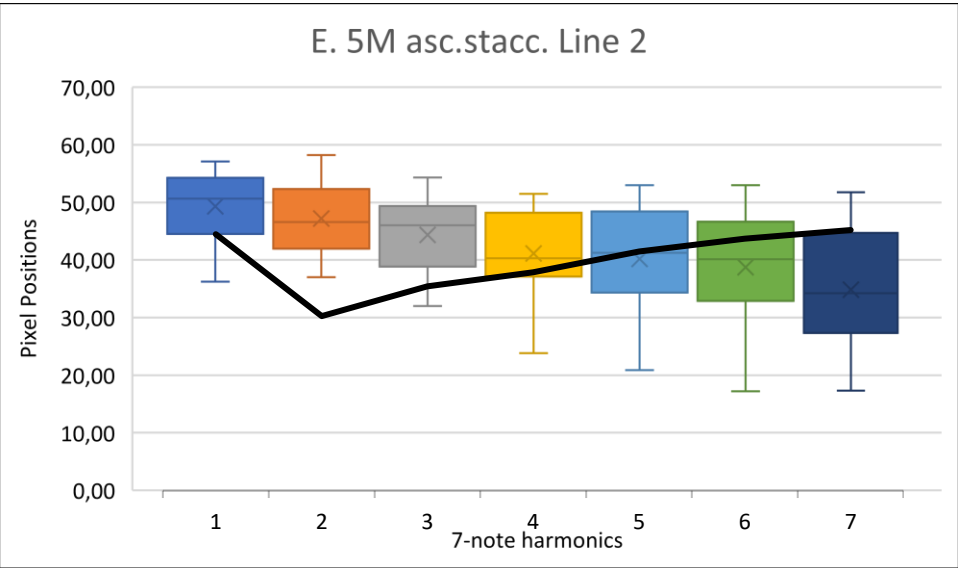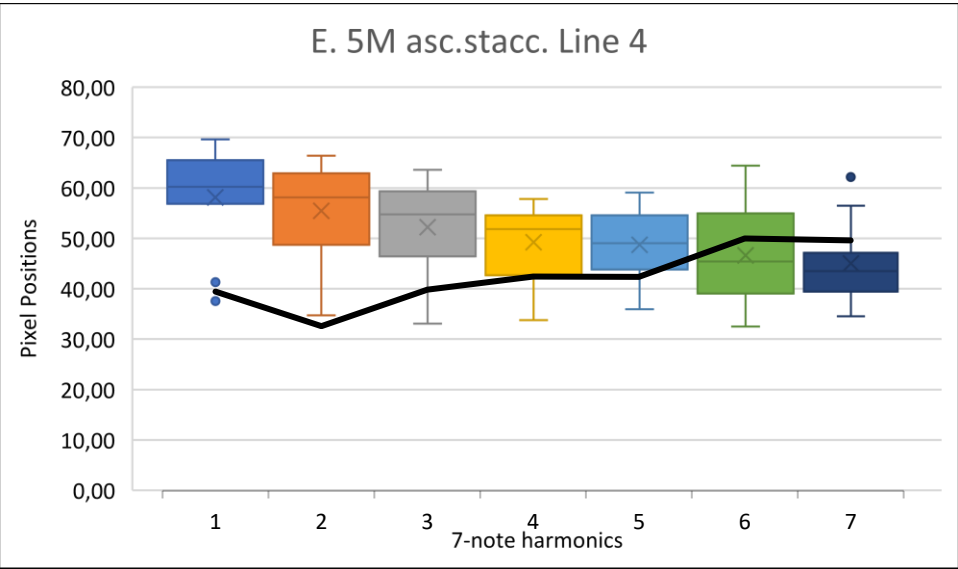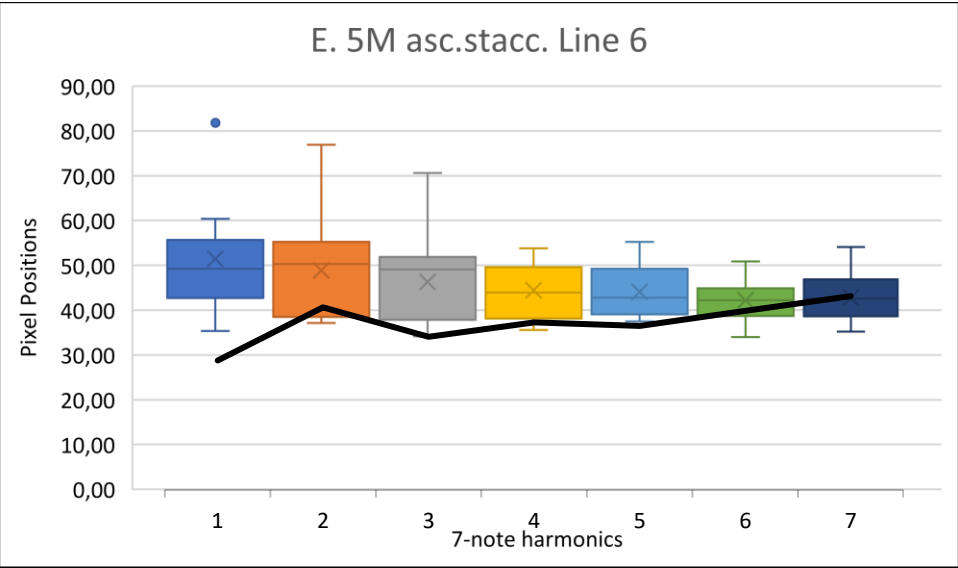

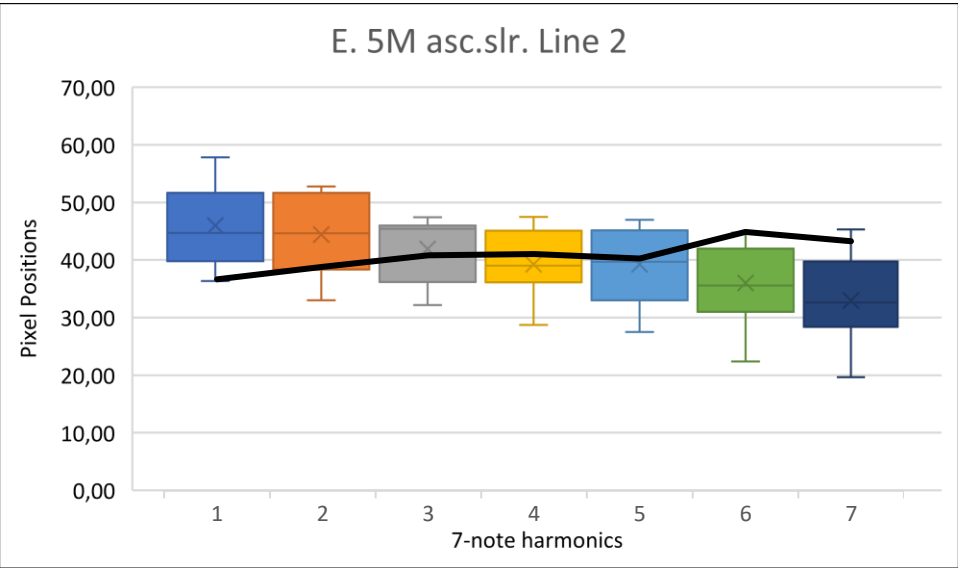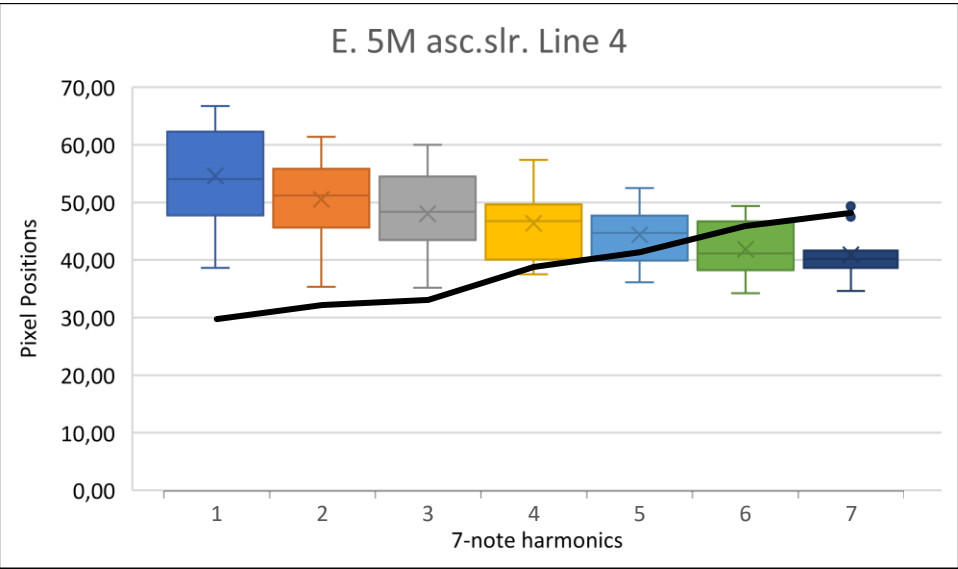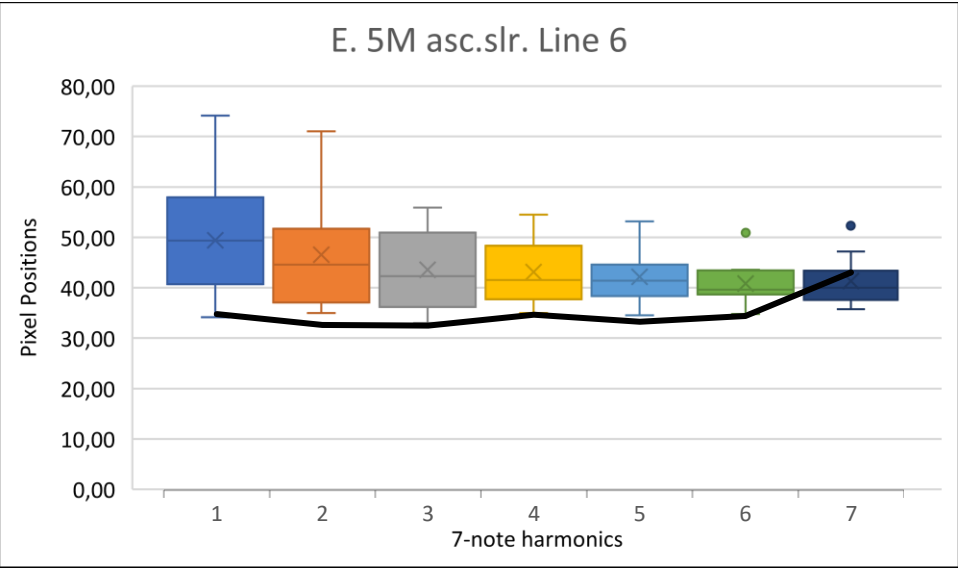

Supplement: SUPPLEMENTARY DATA SHEET 1 — Results of exercise 3M and 5M in ascending and descending slurred and tongued mode as well in staccato. Solid line: Dystonia Patient. [file Data_Sheet_1.PDF]

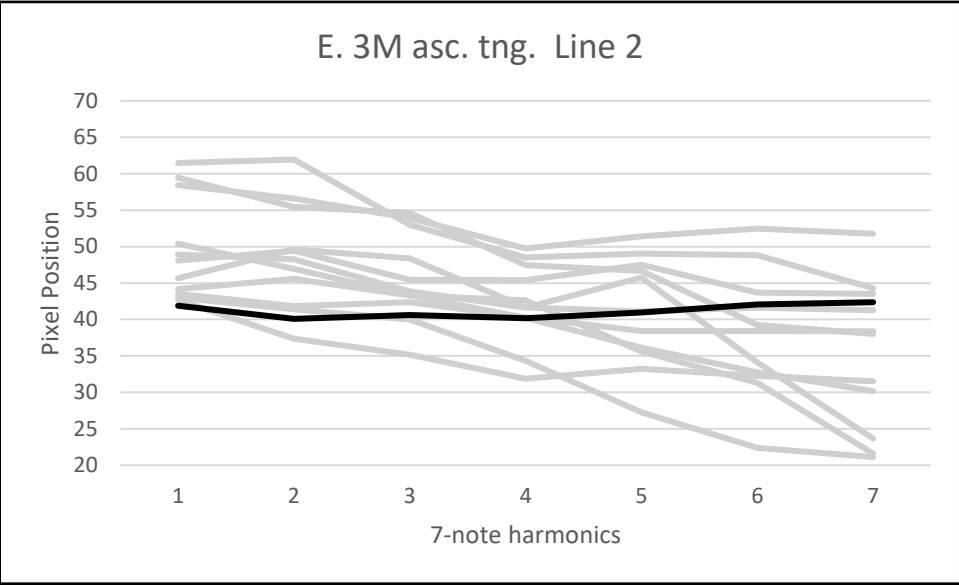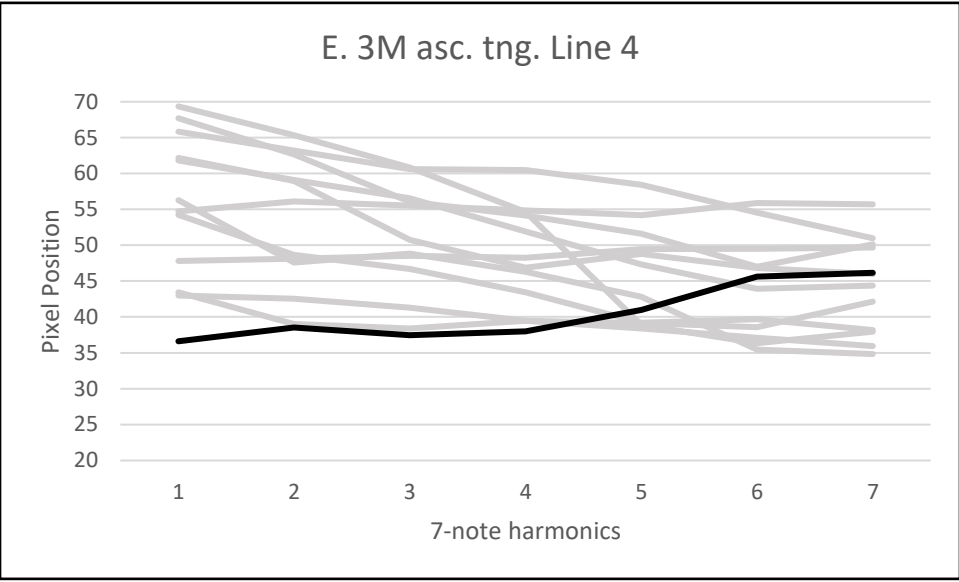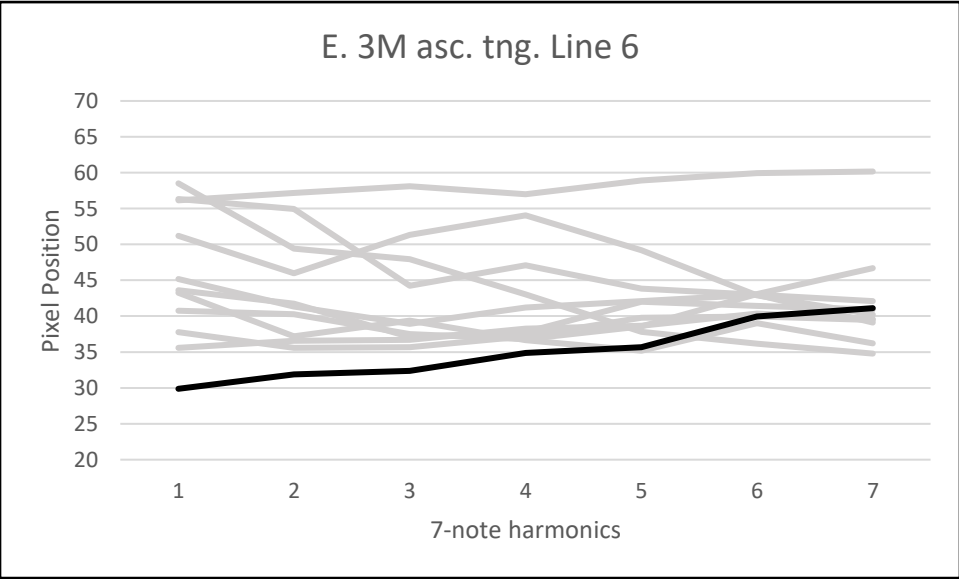

Supplement: SUPPLEMENTARY DATA SHEET 2 — Individual results of exercise 3M, Solid black line: dystonia patient. [file Data_Sheet_2.PDF]
